# Genitourinary Tuberculosis: A Comprehensive Review of a Neglected Manifestation in Low-Endemic Countries

**DOI:** 10.3390/antibiotics10111399

**Published:** 2021-11-14

**Authors:** Guglielmo Mantica, Francesca Ambrosini, Niccolò Riccardi, Enrico Vecchio, Lorenzo Rigatti, Aldo Franco De Rose, André Van der Merwe, Carlo Terrone, Riccardo Bartoletti, Gernot Bonkat

**Affiliations:** 1 Department of Urology, Policlinico San Martino Hospital, University of Genova, 16132 Genova, Italy; f.ambrosini1@gmail.com (F.A.); enrico.vecchio.5@gmail.com (E.V.); aldofrancoderose@gmail.com (A.F.D.R.); carlo.terrone@med.uniupo.it (C.T.); 2 Infectious Diseases Unit, Department of Clinical and Experimental Medicine, Azienda Ospedaliera Universitaria Pisana, University of Pisa, 56126 Pisa, Italy; niccolo.riccardi@yahoo.it; 3 Department of Urology, San Raffaele Hospital, San Raffaele University, 20132 Milan, Italy; rigatti.lorenzo@hsr.it; 4 Department of Urology, Tygerberg Academic Hospital, Stellenbosch University, Cape Town 7505, South Africa; arvdm@sun.ac.za; 5 Department of Translational Research and New Technologies in Medicine and Surgery, University of Pisa, 56126 Pisa, Italy; riccardo.bartoletti@hotmail.com; 6 Alta uro AG, Merian Iselin Klinik, Center of Biomechanics & Calorimetry, University of Basel, 4123 Basel, Switzerland; bonkat@alta-uro.com

**Keywords:** GUTB, genitourinary tuberculosis, tuberculosis, TB, extrapulmonary TB, urogenital TB, neglected diseases

## Abstract

Genitourinary tuberculosis (GUTB) represents a disease often underestimated by urological specialists, particularly in settings such as the European one, where the pathology is less frequent. Similar to other uncommon diseases at these latitudes, GUTB is a neglected clinical problem. In this light, the aim of this review is to give a comprehensive overview of GUTB in order to provide a useful tool for urologists who seldomly manage this disease. A non-systematic review of genitourinary tuberculosis was performed on relevant articles published from January 1990 to July 2021 using PubMed, Scopus, and the Cochrane Central Register of Controlled Trials. GUTB represents up to a quarter of extrapulmonary tuberculosis (EPTB) cases. Diagnostic, therapeutic and surgical work-up have been deeply reviewed and summarized. The mass migration of refugees to Europe as well as the ease of international travel is gradually leading to an upsurge in urological diseases such as GUTB, which were previously only rarely encountered in some European countries. The poor TB knowledge of European urologists should be improved through medical education courses, webinars or telematic means.

## 1. Introduction

Tuberculosis (TB) remains one of the most serious public health problems in the world, representing one of the ten major causes of death [1]. In 2019, the WHO reported around 1.4 million TB deaths and over 10 million new infections [1]. Developing countries are those most seriously affected by the disease *Mycobacterium tuberculosis* (MTB) [2,3,4], but 6% of the global incidence of TB affects Europe, with increasing rates of drug-resistant TB (DR-TB) [3]. Overall, about 500,000 TB patients are coinfected with human immunodeficiency virus (HIV) and another 500,000 develop drug-resistant DR-TB [3].

TB can affect any organ, though the most prevalent and contagious is the pulmonary form (PTB). However, up to 45% of patients may have extrapulmonary involvement (EPTB) [2]. The urogenital tract is one of the most common sites of EPTB, with the urinary tract (kidney, ureter, bladder, urethra) more commonly affected than the genital organs [2,3,4].

Genitourinary tuberculosis (GUTB) represents a disease often underestimated by urological specialists, particularly in settings such as the European one, where the pathology is less frequent [5,6]. Similar to other uncommon diseases at these latitudes, GUTB is a neglected clinical problem. Its symptoms might be mistaken for other urological diseases, and therefore its diagnosis might be delayed. This might lead to irreversible organ damage, with both a worse prognosis for the patients and higher costs for the healthcare system. Furthermore, recently published experiences suggest that the COVID-19 pandemic has had an impact on TB patient care in terms of higher diagnostic delay, reduction in hospitalization, and a greater severity of clinical presentations [7].

In this light, the aim of this review is to give a comprehensive overview of GUTB in order to provide a useful tool for urologists who seldomly manage this disease.

## 2. Materials and Methods

A non-systematic review of genitourinary tuberculosis (Search string: “Genitourinary” AND “Tuberculosis”) was performed in July 2021 on relevant articles using PubMed, Scopus and the Cochrane Central Register of Controlled Trials. The review focused on epidemiology, etiology, physiopathology, diagnosis and management of GUTB. Three authors (GM, FA, EV) independently screened the titles and abstracts of records for eligibility. Articles published after 1990 were taken into consideration, for a total of 3127 manuscripts screened. Reviews, original articles and case reports were included, while other types of articles were excluded. Evidence was limited to human data, and therefore data from animal studies were excluded in the review. Only publications in English were considered. Furthermore, manuscripts not focused on review purposes were not considered. The initial list of selected papers was further enriched by individual suggestions from the co-authors of the present review. Similarly, articles published before 1990 but considered interesting for the purpose of the review were proposed by the authors and evaluated by the screening team. Reference lists of the selected articles/systematic reviews/metanalyses were also screened in order to identify other possible relevant studies using the same criteria of the initial search.

## 3. Results

### 3.1. Epidemiology

GUTB represents up to a quarter of worldwide EPTB cases. The frequency of GUTB depends significantly on geographical and development elements, with more than 90% of GUTB cases occurring in developing countries [8]. In these regions, GUTB is considered the second cause of EPTB, and only about one-third of EPTB patients have a previous diagnosis of TB. HIV is often a concomitant disease in patients with GUTB, especially male patients, who are affected at a rate twice that of than women [8]. In 2019, about 50,000 cases of TB were reported in Europe, resulting in a notification rate of 9.6 per 100,000 population in the EU [9]. Regarding age, the median age of patients affected by GUTB is around 40 years. However, even if it is a typical disease of the adult population, due to its long latency period, it has also been reported in pediatric ages [10].

### 3.2. Aetiology and Risk Factors

GUTB is caused by mycobacteria, of which the most frequently isolated species in humans is MTB *hominis* complex (responsible for about 90% of cases); *Mycobacterium tuberculosis*, *bovis* and *africanum* (especially in West Africa) are the most frequently isolated bacteria. GUTB is almost always secondary due to the hematogenous spread of chronic latent pulmonary TB (LTBI); therefore, primary and LTBI represent its most important risk factors [11,12,13]. Diabetes, old age, low body mass index, oncological comorbidities, immune suppression and renal failure may increase the risk of reactivation of dormant bacilli. This risk of reactivation is estimated to be up to 15% during one’s lifetime [14]. Finally, geographical and social conditions can be considered risk factors. GUTB is more frequent in developing countries and in communities where living conditions of high population concentration and poor hygiene are present.

### 3.3. Physiopathology

TB infection starts from inhalation of cough-generated aerosols containing mycobacterium tuberculosis. When *M. tuberculosis* bacteria enter the alveolar space, they are phagocytosed by alveolar macrophages [15]. In some patients, mycobacteria are totally destroyed by the innate immune system, while in others, they start to replicate into alveolar macrophages [15]. The mycobacteria could spread in other organs or could remain latent in lungs or lymph nodes. GUTB can result from a primary pulmonary infection or from reactivation of an old infection, even after decades [16,17,18]. The principal means by which GUTB develops is via hematogenous spread, reaching multiple organs. The kidney, epididymis and prostate may be sites of infection and disease. Penile TB can also be acquired during sexual contact with infected partners [19]. The upper urinary tract and bladder can be damaged by mycobacteria whenever the disease spreads with the urine, secondary to kidney infection. Prostatic TB has been described also as an adverse event after Bacillus Calmette-Guerin (BCG) intravesical instillation therapy, but it is not covered in this review [20]. In sexually active men, epididymal TB is the most common form of GUTB.

### 3.4. Clinical Presentation

When *M. tuberculosis* reach the kidneys, cortical renal lesions are formed, which result in scarring. Subsequently, after a latent period, reactivation occurs, and the infection generally progresses from a single focus. Often, renal TB is asymptomatic, even though it heavily damages the kidney. Papillary necrosis, calcifications, caseous lesions and parenchymal destruction can be associated with GUTB. Furthermore, if both kidneys are affected, renal failure can occur [21,22]. These papillary lesions caseate and cavitate, forming ulcerocavernous lesions as they erode into the pelvicalyceal system. The ureter, bladder and genital organs are involved by a contiguous spread from the collecting system [23]. Multiple strictures and stenosis generally develop in ureters, with prevalence at the vesicoureteral and uretero-renal junctions [24]. Bladder TB often starts as an acute inflammation process from ureteral meatus with hyperemia and ulceration. Without any treatment, it will result in bladder wall fibrosis and a contracted bladder. Symptoms generally start when the bladder becomes involved. Hematuria, increased urinary frequency and difficulty voiding, as well as abdominal, lumbar and suprapubic pain are the most frequent symptoms. Men may present with penile ulceration and a scrotal/epididymal mass. Women may show menstrual irregularity and pelvic pain [2,4].

### 3.5. Diagnosis and Differential Diagnosis

The diagnosis of GUTB is challenging, since it lacks specific symptoms or signs. Non-specific lower urinary tract symptoms, abnormalities in semen or urine analysis and “sterile” pyuria and/or hematuria could be the first findings of GUTB. A past medical history of TB plays a crucial role in the diagnostic work-up of GUTB, which may have a latency from the pulmonary manifestation of more than 30 years in some cases [25].

#### 3.5.1. Smear Microscopy

The diagnosis is established by the isolation of acid-fast bacilli (AFB) in urine samples, semen, tissue specimens, pus, or discharged or prostatic massage fluid, through microscopic examination using Ziehl–Neelsen (ZN) or auramine staining. AFB smear microscopy of the urine is a rapid test, with 97% specificity but only approximately 20% sensitivity [26].

#### 3.5.2. Urine Culture

At least three early-morning urine samples, delivering first-void midstream, on consecutive days, are recommended for AFB culture. In general, the culture-based method for urine or tissue biopsy specimens is the gold standard, with a sensitivity of 80–90% and a specificity of roughly 100%. Moreover, it could concurrently provide data on TB drug susceptibility [26]. The disadvantage of culture-based methods is the time needed for the results. The liquid culture system, which is recommended as the diagnostic gold standard by the WHO, takes at least 9–10 days for positive results and 6 weeks to be considered negative.

#### 3.5.3. Nucleic Acid Amplification Tests

In recent years, nucleic acid amplification tests (NAATs) have been introduced in the diagnostic pathway of TB, to overcome the limits of early and rapid diagnosis and of drug susceptibility testing. Currently, NAATs such as RT-PCR (Gene Xpert MTB/RIF by WHO in 2010 or GeneXpert MTB/RIF ultra by WHO in 2017) are recommended for the detection of pulmonary TB as, according to the latest WHO policy updates of 2013, these technologies cannot be routinely applied on urine samples. They are promising technologies for detecting *Mycobacterium* DNA (Mbt DNA) in urine samples requiring further validations [27,28].

#### 3.5.4. Whole-Genome Sequencing (WGS)

WGS can provide the complete genome of *M.* spp. in a sample, giving information such as drug-resistance and the transmission patterns. WGS can be very useful in providing particular information to build more effective and safer anti-TB regimens [29].

#### 3.5.5. Histological Examination

The histopathological examination of tissue specimens collected from biopsies or fine-needle aspirates is helpful to detect granulomas and to identify mycobacteria. In some cases, the pathological report is the only chance to yield diagnosis, as in case of TB involving epididymis [30]. Differential diagnosis of granulomas includes a wide range of infectious diseases and non-infectious diseases [31].

#### 3.5.6. Imaging

The positivity of laboratory assays does not reveal the site of GUTB nor the impact on the genitourinary system. Thus, radiological imaging plays a fundamental role in localizing the foci of the disease and the extent of the damage. The classical radiological findings are historically based on conventional radiography and intravenous urography (IVU). Currently, computed tomography (CT), Magnetic Resonance Imaging (MRI) and ultrasonography (US) are more frequently performed to yield a diagnosis. Moreover, imaging technologies could be applied for targeting biopsies. Overall, imaging techniques are up to 91.4% sensitive for GUTB [32].

##### Plain X-ray

Plain abdomen and chest X-rays are currently recommended in all cases of TB suspicion to identify any site of active or healed TB disease [33]. The most common sign of TB is calcification of affected organs, such as the lungs and kidneys. Several differential diagnoses should be considered, such as helminth infections, neoplasms and abscesses [34].

##### Intravenous Urography (IVU)

Although IVU has been superseded by CT intravenous urography (CT IVU), it can provide imaging that may help in GUTB diagnosis. In the early stage of kidney TB, mucosal oedema and irregularity of the excretory system can be detected [35]. Ureter involvements may show lumen irregularity or strictures/stenosis. Bladder TB may show an organ with wall thickening and reduced capacity. One of the main concerns regarding IVU is the limited visualization of the upper urinary tract in obstructive uropathy.

##### Ultrasonography (US)

US may be useful for studying the upper urinary tract in the case of TB suspicion, although it can return negative results in the early stage of disease. Renal TB might show abscesses, hypoechoic lesions or hydroureteronephrosis caused by ureteral strictures. In the later stages, calcification can be identified within the kidney. The differential diagnosis for calcification includes renal schistosomiases, hydatid cysts and renal abscesses [36,37]. A bladder affected by TB may appear as a low-capacity organ with a thick wall. Prostate TB may show multiple hypoechoic areas peripherally, mimicking prostate cancer. Transrectal US might be useful in detecting related complications such as prostatic abscess [38]. In the case of the suspected involvement of testes, epididymis or vas deferens, US represents the diagnostic gold standard. In these cases, US shows diffuse or localized hypoechoic swelling and calcifications [39]. In females with suspected GUTB, US may show ovarian masses. Patients with endometrial TB show intrauterine thickening, and in later stages, calcifications could be detected [40]. US is an important tool, especially in developing countries. In fact, it has a relatively reasonable learning curve and is ionization-free, portable and increasingly available at reasonable costs [41].

##### CT Scan and CT Urography

**Figure 1:**
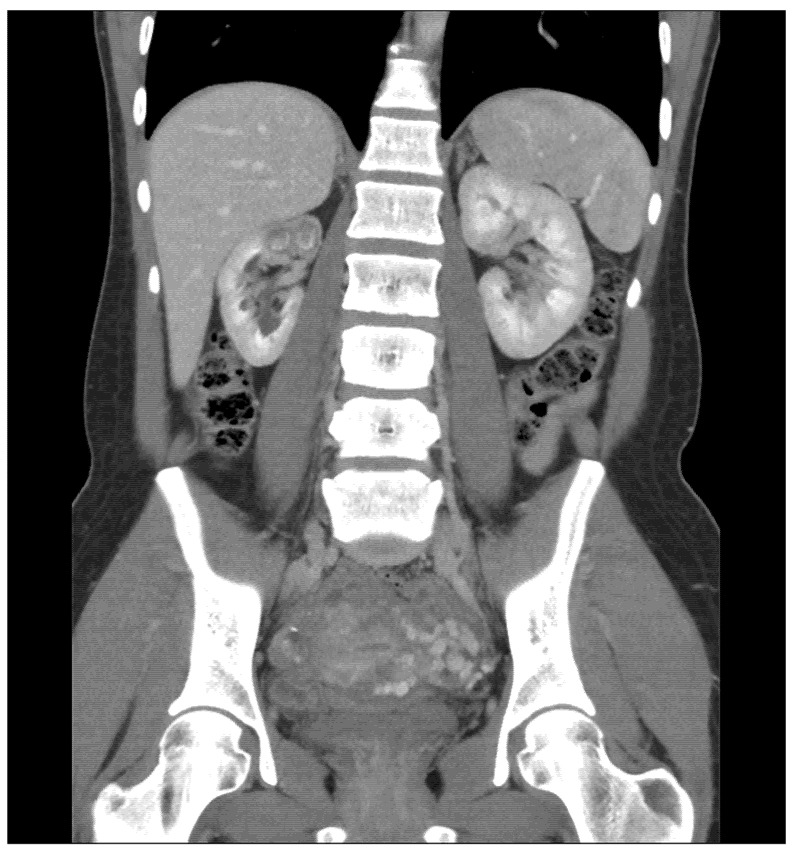
CT Scan image showing right upper pole kidney TB lesions—coronal view.

**Figure 2:**
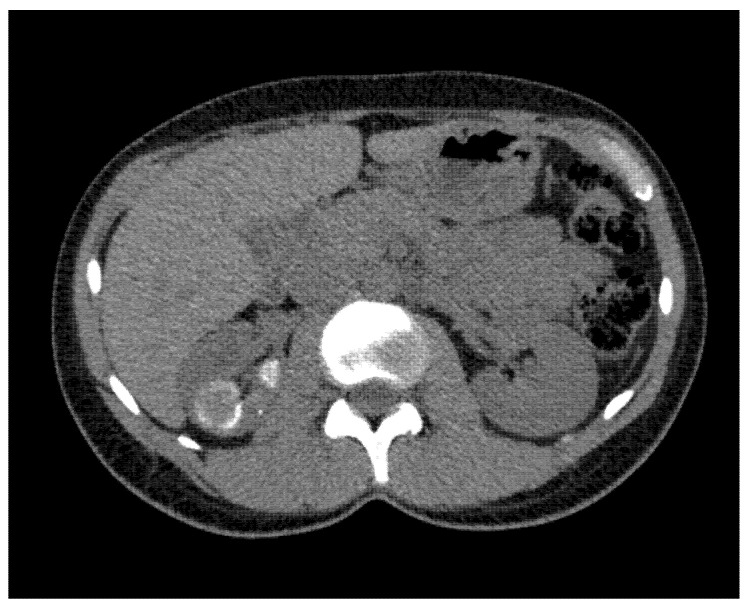
CT Scan image showing right upper pole kidney TB lesions—transversal view.

**Figure 3:**
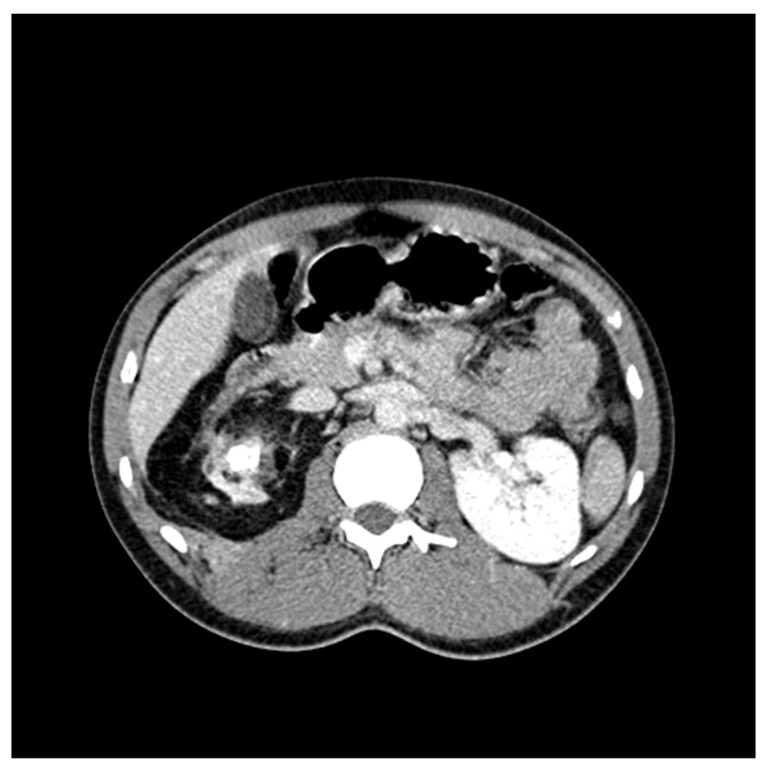
CT Scan showing perinephric edema mimicking pyelonephritis.

**Figure 4:**
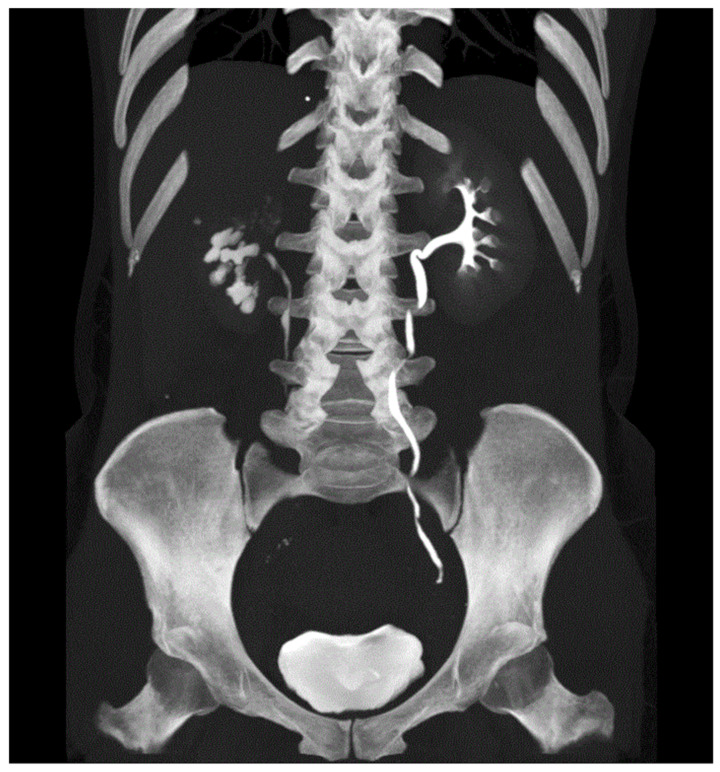
CT IVU showing right kidney delayed excretion and left ureter with multiple kinking and stenosis.

CT scan is able to detect clear signs of GUTB. When the renal parenchymal is involved, CT urography may show granulomas during nephrogenic phases. If larger in size, the granulomas could mimic a renal mass or a complex cystic lesion [42]. Sometimes, the findings may also be confused with pyelonephritis or renal abscesses. Parenchymal renal TB can evolve into fibrosis and scars. Moreover, calcifications could be found up to a complete replacement of the renal parenchyma with calcific tissue (auto-nephrectomy). Typical images of renal TB are lobar calcifications, with calcified rims outlining damaged renal lobes [33]. Cavities and excavations can be detected as early signs of papillary necrosis (Figure 1 and Figure 2) [43]. Differential diagnosis with transitional cell carcinoma (TCC) or with xanthogranulomatous pyelonephritis can be challenging (Figure 3). The features of calcifications can help in the diagnosis. If the pyelo-ureteral junctions are involved, hydronephrosis can be detected. Antegrade spread of TB can affect the ureter, bladder and urethra. The distal third of the ureter is the most common site of damage. Ureteral thickening, ulcerations and calcifications, resulting in ureteral strictures and hydronephrosis, are the most common signs of ureteral involvement (Figure 4). Concomitant ureteral granulomas could result in filling defects, which can be confused with TCC [44]. Ureteral calcification could mimic schistosomiasis, which generally is intramural and is not contiguous with the renal collecting system [45]. Bladder TB generally presents with wall thickening and calcification, as well as mucosal ulcerations up to contracted fibrotic obliteration. The male genital tract can be involved, and differential diagnosis with sexually transmitted infections such as gonorrhea and syphilis should be considered. During the acute phase, seminal vesicles can appear enlarged, with wall and septal thickening at CT. Atrophy and calcifications can be observed during chronic disease. These non-specific signs must be distinguished from other forms of bacterial seminal vesicle inflammation, such as schistosomiasis, in particular in endemic countries [46]. In the case of female GUT, the fallopian tubes are the most frequently affected organs, accounting for 90% of cases. This presents with enlargement, hydrosalpinx, pyosalpinx, and wall thickening, with calcification at CT [47]. Cervical TB may show heterogeneous enhancing at imaging, with distorted anatomy. Histopathological examination is necessary to exclude carcinoma [40].

##### Positron Emission Tomography (PET)–CT Imaging

18F-fluorodeoxyglucose Positron Emission Tomography (18F-FDG-PET) is not routinely used; while it can identify sites of active inflammatory disease, it cannot differentiate GUT from cancer [48].

##### Magnetic Resonance Imaging (MRI)

MRI has low sensitivity for the detection of GUT in the early stage of disease. It was first used for the evaluation of renal TB. MRI can be useful in pediatric patients and during pregnancy, to avoid exposure to radiation. Renal abnormalities are comparable to those described for CT findings and must be distinguished from acute pyelonephritis [49]. The multiparametric magnetic resonance imaging (mp-MRI) of the prostate is the most useful diagnostic technique to distinguish the nodular or diffuse patterns of prostate TB. The nodular type has low T2 signal intensity in MRI, with diffusion restriction and DCE. The diffuse type can be detected as low streaky lesions in the peripheral zone at T2 with weighted MRI [50]. Using MRI, TB lesions of testis and/or epididymis, vas deferens and/or spermatic involvement could appear T2-hypointense, suggesting chronic inflammation and fibrosi [39]. Furthermore, MRI could be useful for the evaluation of the female genital tract, for example, for the diagnosis of abdominal adnexal masses. Enhancing nodules or masses of the vagina or the vulva could be described [51].

##### Endoscopy–Laparoscopy

Diagnostic endoscopic procedures, including cystourethroscopy, ureteroscopy, hysteroscopy and laparoscopy, are useful to detect TB lesions and even to obtain biopsy specimens of abnormal tissue. Cystoscopy could reveal generic signs such as hyperemia, granulomas and ulcerations. Bladder biopsy is 18.5% to 52% sensitive [32]. In women with GUT and infertility, laparoscopy should be recommended to evaluate the peritoneum cavity, the fallopian tubes and the ovaries, and biopsies should be obtained. Hysteroscopy should be considered to explore endometrial integrity, distortion of the uterine cavity, and tubal ostia. In the case of suspected male urethral involvement, although it is uncommon, foci of disease could be noted as ulcerations or, finally, strictures at endoscopy or urethrography. Complicated urethral TB could result in fistulas into the perineum or surrounding areas, which can be detected with cystourethrography [32]. Differential diagnosis includes schistosomiasis, balanitis xerotica obliterans, fungal infections and chronic bacterial infections [52].

##### Hysterosalpingography (HSG)

HSG is the imaging of choice for the evaluation of the anatomy of the female genital tract, especially the fallopian tubes. Some authors use laparoscopy for investigating the abnormalities of the female genital tract, although HSG remains the gold standard to assess the fallopian tube patency [53]. Tubal obstruction is the most common finding with HSG. The presence of a convoluted fallopian tube may suggest pelvic peritoneal adhesions disrupting the regular anatomy. Nonspecific deformity of the uterine cavity can be observed, such as a T-shaped uterus, resulting from abnormal scaring and fibrosis. This process could lead to a complete obliteration of the uterine cavity (“Netar syndrome”) [54].

### 3.6. Medical Treatment

The WHO recommends a 6-month regimen for GUTB, including an intensive phase of 2 months with isoniazid (5 mg/kg every 24 h; max dosage 300 every 24 h), rifampicin (10 mg/kg every 24 h; max dosage 600 every 24 h), pyrazinamide (25 mg/kg every 24 h; max dosage 2000 every 24 h), and ethambutol (15–20 mg/kg every 24 h), followed by a continuation phase of 4 months with either isoniazid and rifampicin. The long duration can affect the adherence, which is essential to achieve successful cure rates as well as to prevent relapses [55]. A 4-month rifapentine-based regimen containing moxifloxacin was found to be noninferior to the standard 6-month regimen in the treatment of pulmonary TB, but for extrapulmonary TB, data are still lacking [56]. Fluoroquinolone-based shortening trials showed that earlier sputum culture conversion may not be a reliable surrogate of earlier cure rates. Other trials have evaluated repurposed (i.e., clofazimine, linezolid) or new drugs (i.e., bedaquiline), currently recommended for DR-TB patients [57,58]. The pattern of resistance of the MTB isolate, and thus certainty of the cure definition, such as persistent radiological lesions [59] and/or persistent clinical symptoms, may involve prolonged anti-tubercular treatment. In fact, microbiological, immunological and radiological biomarkers, always coupled with urological treatment, should be used to stratify patients needing a tailored therapeutic approach with a more prolonged treatment when necessary [60]. Drug resistant TB therapy is based on long regimens, associated with a high frequency of adverse events (up to ~60%) and poor clinical outcomes (about 30% mortality rate) [61,62]. A shorter regimen, whose effectiveness could be monitored through clinical, radiological and microbiological markers, is needed. Moreover, effective oral drugs with a favorable drug–drug interaction profile might be the most convenient approach to improve patient adherence and favor outpatient follow-up in the case of urogenital TB [63,64]. The appropriateness of corticosteroid treatment in patients with GUTB at high risk of developing ureteral strictures is still debated. Some published cases show how ureteral stricture may relieve drastically after starting an oral corticosteroid regimen. Pre-emptive administration of corticosteroids may be effective in preventing further deterioration of the ureteral stricture in patients with a pre-existing ureteral lesions [65]. Consultation with reference centers for the managing of DR-TB is highly recommended in order to build the most effective regimens.

### 3.7. Surgical Management

In uncomplicated patients, the standard of care is medical treatment. The diagnosis of GUTS is often delayed due to both the non-specific initial symptoms and its insidious and slow progression, leading to a high rate of urogenital organ impairment. In more than 50% of patients affected by GUTB, surgery is recommended, with an ablative, endoscopic or reconstructive approach because of the caseous or fibrosing reaction, which slowly destroys the genitourinary system [66]. There is still considerable controversy regarding the best surgical treatment, and a standardized surgical plan does not exist. The heterogeneity of the disease presentation and the different classification systems, which are not generally accepted, may explain this issue. The optimal timing for surgery is controversial. Some authors suggest a delay of 2–6 weeks after the initiation of medical treatments, up to 9 months in some cases. This strategy makes it possible to reduce active inflammation and to stabilize lesions [67]. The endoscopic techniques include strategies to restore the patency of the urinary tract, such as internal urethrotomy, urethral dilatation and endopyelotomy. In patients presenting with hydronephrosis due to vesicoureteral junction, ureteral, or pelvic ureteral junction obstruction, drainage by ureteral stent of nephrostomy should not be postponed, preventing renal failure. Subsequently, medical treatment is recommended before pre-operative revaluation [67]. In the case of persistent urogenital deformity, the patient may be submitted to a definitive surgical treatment [68]. The ablative treatments are aimed at removing the TB foci. In general, patients presenting with non-functioning symptomatic renal TB, complicated by hypertension or diffuse renal lesions, should undergo a radical surgery. According to the research of Kerr et al., nephrectomy should be recommended even in case of asymptomatic non-functioning kidneys, to avoid the reactivation of disease renal foci [24]. The literature lacks strong evidence regarding the recommendation on partial nephrectomy in the case of a single, small TB lesion. Although some series on this topic are available, most of the authors agree that nephrectomy should be always be considered because of the risk of reactivation of misdiagnosed renal foci [69]. With regard to the best surgical approach, a consensus does still not exist. The minimally invasive options, such as laparoscopic, single-site laparoscopic, and retroperitoneoscopic techniques, have been reported as feasible and safe strategies, comparable to open surgery [70,71,72]. Reconstructive surgery is recommended to repristinate the normal genitourinary functions and may include augmentation cystoplasty, uretero-ureterostomy, ureteroneocystostomy, ureteral reimplant, pyeloplasty, ureterocalicostomy and ileal ureter or external diversion, where indicated [73]. In particular, in the case of a contracted bladder, with a low capacity of approximately 150–200 mL, augmentation cystoplasty could be considered to obtain a low-pressure voiding system. If the bladder anatomy is severely impaired, with a reduced capacity of 15–20 mL, an orthotopic neobladder following cystectomy may be a feasible option [74].

### 3.8. Follow-up

Long-term monitoring is often limited by the difficulty in collecting tissue specimens. Thus, the response to treatments is generally evaluated through radiological and clinical findings. The severity of the baseline condition, the site involved, and the treatments received influence the follow-up plan. No standard recommendations regarding the most effective follow-up protocol exist, and the end of the treatment remains controversial. Pulmonary TB may result in relapse in 2% to 6% of cases, particularly within the first year after treatment. GUTB patients may relapse at a higher rate than pulmonary TB, in 6.3% to 22% of cases, even after 12 months of medical therapy [75]. One of the possible explanations for the higher relapse rate may lay in the innumerable renal TB foci, which are difficult to completely sterilize with medical treatment [75]. Long-term monitoring for GUT patients is recommended up to 10 years, as the average time of relapse is approximately 5 years (range, 11 months to 27 years) [76].

## 4. Conclusions

GUTB diagnosis may be challenging, since it can involve any part of the genitourinary system, and presentation may vary from vague urinary symptoms to chronic kidney disease. The usual tests used to diagnose GUTB are the demonstration of *M.* spp. in urine or body fluids and imaging examination (IVU, US, CT urography, etc.). Newly available tests, such as WGS, can provide the complete genome of *M.* spp. in a sample, giving information such as drug resistance and transmission patterns. In uncomplicated patients, the standard of care is medical treatment. GUTB diagnosis is often delayed, leading to a high rate of urogenital organ impairment, and in more than 50% of patients, to surgical management. Long-term monitoring for GUT patients is recommended up to 10 years.

In 2019, about 50,000 cases of TB were reported in Europe, resulting in a notification rate of 9.6 per 100,000 population in the EU [9]. This may translate to about 2.5 cases of GUTB per 100,000 inhabitants. Therefore, even if GUTB can be considered a rare disease at this latitude, urologists can diagnose and manage such patients. In countries such as Italy, Germany, Greece and Iceland, the foreign origin of TB ranges from 50 to 80% of cases [9]. The mass migration of refugees to Europe and the ease of international travel are leading to an upsurge in urological diseases such as GUTB, which were previously only rarely encountered in some European countries [5]. In 2019, a survey investigating knowledge of diagnosis and management of “tropical diseases” such as schistosomiasis, GUTB and filariasis was distributed to hundreds of European urologists. Regarding GUTB, less than 20% of the surveyed urologists showed satisfactory knowledge [5]. Misdiagnosis and incorrect management of these conditions by urologists in European countries are likely to lead to poor utilization of resources and an increase in healthcare costs in the future. What can use we have learned from the recent COVID-19 pandemic to improve health planning, teaching and research towards other infectious diseases that are neglected in much of Europe [77]. The knowledge of European urologists can be improved through medical education courses, webinars or telematic means [78]. If, once the COVID-19 pandemic is over, we direct some of the resources and skills acquired in the management of endemic infectious diseases towards neglected diseases such as GUTB, we will be on the right track to reduce the spread and further improve the management of such pathologies.

Despite the limitations of the current study, this review provides to European urologists a useful and comprehensive tool on the diagnostic and therapeutic treatment of patients with suspected GUTB.
